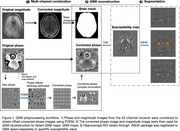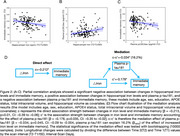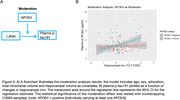# Longitudinal hippocampal iron accumulation predicts episodic memory in presymptomatic Alzheimer's disease with additional influences of tau and APOE geneotype

**DOI:** 10.1002/alz70856_105845

**Published:** 2026-01-07

**Authors:** Jing Zhou, Alfie Wearn, Julia Huck, Colleen S. Hughes, Giulia Baracchini, Elisabeth Sylvain, Jennifer Tremblay‐Mercier, Judes Poirier, John C.S. C. S. Breitner, Sylvia Villeneuve, Mallar M. Chakravarty, Christine L Tardif, Claudine J Gauthier, Ana M. Daugherty, Gary R. Turner, R. Nathan Spreng, for the PREVENT‐AD Research Group

**Affiliations:** ^1^ Montreal Neurological Institute, McGill University, Montreal, QC, Canada; ^2^ Université de Sherbrooke, Sherbrooke, QC, Canada; ^3^ Indiana University, Bloomington, IN, USA; ^4^ Brain and Mind Centre, The University of Sydney, Sydney, NSW, Australia; ^5^ StoP‐AD Centre, Douglas Mental Health Institute Research Centre, Montreal, QC, Canada; ^6^ Centre for Studies on Prevention of Alzheimer's Disease (StoP‐AD Centre), Montreal, QC, Canada; ^7^ Department of Psychiatry, McGill University, Montreal, QC, Canada; ^8^ Department of Psychiatry, McGill University, Montréal, QC, Canada; ^9^ Cerebral Imaging Centre, Douglas Mental Health Institute Research Centre, Montreal, QC, Canada; ^10^ Department of Biomedical Engineering, McGill University, Montreal, QC, Canada; ^11^ McConnell Brain Imaging Center, McGill University, Montreal, QC, Canada; ^12^ Montreal Heart Institute, Montreal, QC, Canada; ^13^ Concordia University, Montreal, QC, Canada; ^14^ Wayne State University, Detroit, MI, USA; ^15^ York University, Toronto, ON, Canada

## Abstract

**Background:**

Elevated brain iron deposition is recognized as a characteristic of normal aging and neurodegenerative diseases, particularly Alzheimer's disease (AD), where it correlated with amyloid‐β plaques and neurofibrillary tangles. Our study aimed to investigate the relationship between longitudinal changes in hippocampal iron deposition and episodic memory, and how this relationship is impacted by AD pathology and APOE4 allele carriership.

**Method:**

We measured longitudinal changes in brain iron levels using quantitative susceptibility mapping (QSM)‐MRI (see Figure 1), in a cohort of old adults at risk of AD (*N* =143, 102 females, 41 males; mean age = 67.7 ± 5.0 years; longitudinal duration = 2.7 ± 0.4 years). Cognition was assessed using the RBANS. Plasma was collected from all participants at a single time point (Time 2, T2) and *p*‐tau181 measured using in‐house single‐molecule arrays. We examined the relationship between iron accumulation and memory, the mediating effect of plasma *p*‐tau181. We also investigated how APOE4 status moderates the relationship between iron deposition and plasma *p*‐tau181.

**Result:**

Hippocampal iron levels demonstrated a significant increase over time (t(142)=2.45, Cohen's d=0.21, *p* = 0.016). Changes in iron levels were significantly negatively correlated with memory performance (β=‐0.223, *p* = 0.009, Figure 2A), and positively associated with plasma *p*‐tau181 (β=0.217, *p* = 0.011, Figure 2B). Plasma *p*‐tau181 were also negatively associated memory (β=–0.207, *p* = 0.015, Figure 2C). Furthermore, *p*‐tau181 mediated the relationship between hippocampal iron increases and memory performance, accounted for 16.2% of the total association (β = −0.034, *p* = 0.045, CI: −0.09 to ‐0.004, Figure 2D). APOE4 status moderated the impact of increased hippocampal iron on plasma *p*‐tau181 levels (β =0.431, *p* = 0.021, CI: 0.06 to 0.8, Figure 3).

**Conclusion:**

These findings underscore the unique effect of hippocampal iron accumulation on cognition, which is additionally impacted by AD pathology. Further, we find a novel association in APOE4 carriers, wherein increases in iron interact with AD pathology, which highlights the need for early detection and intervention strategies tailored to APOE4 carriers. This work deepens our understanding of the interplay among iron dysregulation, tau pathology, and APOE4, offering a promising avenue for precision‐based approaches to AD risk assessment and therapeutic development.